# Electrochemically Treated TiO_2_ for Enhanced Performance in Aqueous Al-Ion Batteries

**DOI:** 10.3390/ma11112090

**Published:** 2018-10-25

**Authors:** Alexander Holland, Rachel McKerracher, Andrew Cruden, Richard Wills

**Affiliations:** Energy Technology Research Group, University of Southampton, Southampton SO17 1BJ, UK; awh1g10@soton.ac.uk (A.H.); R.D.McKerracher@soton.ac.uk (R.M.); A.J.Cruden@soton.ac.uk (A.C.)

**Keywords:** aqueous Al-ion, battery, high-rate, electrochemical treatment, titanium dioxide

## Abstract

The potential for low cost, environmentally friendly and high rate energy storage has led to the study of anatase-TiO_2_ as an electrode material in aqueous Al^3+^ electrolytes. This paper describes the improved performance from an electrochemically treated composite TiO_2_ electrode for use in aqueous Al-ion batteries. After application of the cathodic electrochemical treatment in 1 mol/dm^3^ KOH, Mott–Schottky analysis showed the treated electrode as having an increased electron density and an altered open circuit potential, which remained stable throughout cycling. The cathodic treatment also resulted in a change in colour of TiO_2_. Treated-TiO_2_ demonstrated improved capacity, coulombic efficiency and stability when galvanostatically cycled in 1 mol·dm^−3^AlCl_3_/1 mol·dm^−3^ KCl. A treated-TiO_2_ electrode produced a capacity of 15.3 mA·h·g^−1^ with 99.95% coulombic efficiency at the high specific current of 10 A/g. Additionally, X-ray diffraction, scanning electron microscopy and X-ray photoelectron spectroscopy were employed to elucidate the origin of this improved performance.

## 1. Introduction

Aqueous intercalation batteries are being explored as potentially cheap, safe, non-toxic and high power energy storage devices [1,2,3,4]. Aqueous Al-ion batteries may provide a promising chemistry due to the low cost and high abundance of aluminium [5,6]. Anatase-TiO_2_ is one of only a few negative insertion electrode materials shown to function in aqueous electrolytes and has shown some promising performance characteristics with regard to power capability in aqueous aluminium containing electrolytes [6,7,8,9,10,11]. Previous publications have attributed charge storage to the reduction of Ti^4+^ to Ti^3+^ with the concomitant intercalation of Al^3+^ [6,7,9,10], though surface charge storage via a capacitive or psuedocapacitive mechanism cannot be ruled out. Nevertheless, charge storage has been shown to be due to the presence of Al^3+^ and not H^+^, K^+^ or Na^+^ in aqueous chloride electrolytes [5,11]. Other insertion materials with a suitably negative potential range in aqueous electrolytes, excluding those based on lithium salts, are limited to Nasicon titanium phosphates and vanadium oxides, while activated carbon has also been employed as a capacitive negative electrode [12,13,14,15]. NaTi_2_(PO_4_)_3_, for use as a negative electrode in aqueous Na-ion, seems to be promising. A capacity of 60 mA·h·g^−1^ at 2.66 A/g was measured by Hang et al. [16]. The possibility of using a dual-ion electrolyte to construct a full cell, where separate cations are reversibly inserted into the negative and positive electrodes, has been demonstrated, opening the possibility of using a variety of electrode combinations [5,17,18,19]. Therefore, TiO_2_ in aqueous Al^3+^-containing electrolytes provides an opportunity for creating aqueous cells with high working voltages. However, to date there has been limited discussion of the low coulombic efficiency of anatase TiO_2_ in aqueous aluminium salt electrolytes, especially during initial cycles. This is an important point for the electrode to be considered for use in full, two electrode battery cells. Furthermore, methods to improve the performance of TiO_2_ in aqueous aluminium electrolytes have generally focused on nanostructures such as nanospheres, nanotube arrays or the addition of graphene flakes [6,8,10]. A relatively high capacity of ca. 150 mA·h·g^−1^ was measured from TiO_2_ nanospheres at the low specific current of 50.25 mA/g, while a graphene-TiO_2_ electrode produced a discharge capacity of ca. 20 mA·h·g^−1^ at a high current of 6.25 A/g. However, the coulombic efficiency of these TiO_2_ structures is low, with the coulombic efficiency of graphene incorporated TiO_2_ being approximately 50%. 

Doped TiO_2_ could provide a method to incorporate Ti^3+^ to improve the electrodes’ ionic and electronic conductivity whilst potentially improving insertion kinetics of Al^3+^. However, only He et al. have described the potential of doped TiO_2_ via their synthesis of black nanoleaves [7]. Their method of solution plasma processing is not yet well understood and may not be feasible for producing large quantities of material; as such, alternative methods of doping are important to help verify the effect of doping on TiO_2_ electrodes and its application in aqueous batteries [20,21]. 

Other methods of TiO_2_ doping have been widely explored for use in other applications such as photocatalytic hydrogen production, environmental pollutant removal or supercapacitor electrodes [22]. It is with alternative applications in mind that various methods of doping have been proposed. Doped TiO_2_ nanostructures have been prepared via high temperature (> 500 °C) treatments in H_2_, Ar and N_2_ atmospheres [23,24,25,26]. The electrochemical reduction method used has been previously reported as a safe and facile method of producing reduced (self-doped) TiO_2_. Electrochemically doped TiO_2_ nanotube arrays has shown improved electrochemical performance as aqueous supercapacitor current collectors, while self-doped TiO_2_ nano structures have demonstrated improved photocatalytic activity for water splitting [27,28]. Here, we report that this safe, facile and repeatable reduction treatment can be applied to composite TiO_2_ electrodes in order to improve their performance as a negative electrode in aqueous AlCl_3_ electrolyte, demonstrating high rate capability and stability during cycling. Significantly, doping of TiO_2_ is demonstrated to be an important factor for improving coulombic efficiency and initial cycling stability in aqueous electrolyte.

## 2. Materials and Methods 

Composite TiO_2_ electrodes were manufactured through combining TiO_2_ nanopowder (87.5 wt%), Vulcan XC-72 carbon black (5 wt%), Nafion binder (7.5 wt%) and additional propanol (approximately 3 g for every 1 g of powder). The resulting ink was mixed using a Silverson high speed shear mixer for 30 min at 5000 rpm, then sonicated for a further 20 min. TiO_2_ was purchased from US-nano and had a nominal particle size of 5 nm. The resulting ink was then coated onto a carbon polymer current collector, which was used because of its electrochemical stability in acidic electrolytes and for being more cost effective than other metal current collectors, such as titanium or platinum foils and meshes. Electrodes were allowed to dry in ambient conditions overnight, resulting in 23–24 mg of active electrode covering an area of 7 cm^−2^, where the electrode covered 1 cm × 3.5 cm of both sides of the current collector. 

Electrochemical experiments were carried out in 3-electrode cells using a saturated calomel reference (SCE) electrode and copper-hexacyanoferrate as a reversible counter electrode. The electrolyte used was 1 mol·dm^−3^ AlCl_3_/1 mol·dm^−3^ KCl. The electrochemical treatment process consisted of holding electrodes at −1.40 V vs. SCE in 1 mol·dm^−3^ KOH for 15 min, which was repeated ten times with a 3 min open circuit potential (OCP) between repeats. No obvious bubbling or H_2_ evolution was visible during chronoamperometry. Electrodes were left in the KOH electrolyte for several hours before being washed, in de-ionised water, and transferred into a 3-electrode cell with a AlCl_3_/KCl electrolyte. Galvanostatic cycling and electrochemical impedance spectroscopy were performed on a Solartron 1470E battery analyser. Electrochemical impedance spectroscopy (EIS) was performed to produce both Mott–Schottky and Nyquist plots. For Mott–Schottky analysis, EIS was performed using a 10 mV perturbation between 10 kHz and 10 Hz at DC bias potentials between 0.4 V and −0.7 V vs. SCE. For the Nyquist plots, EIS was performed on electrodes in partially charged states, achieved by applying a 100 mA/g charge current to −0.8 V and −0.9 V, holding the electrodes at these potentials for 120 s, then performing measurements at OCP. These measurements were performed between 0.1–10 kHz using a 10 mV perturbation around the OCP. 

X-ray diffraction (XRD) was carried out using a Bruker D2 phaser with Cu Kα radiation (average wavelength = 1.5418 Å). Data points were collected in the 2θ range 15° to 75° with a 0.0202572° increment and 0.4 s time step. Scanning electron microscope (SEM) images were obtained using a JSM 6500F field emission electron microscope, which was operated at 15.0 kV and a working distance of 13.7 mm. 

## 3. Results

### 3.1. Electrode Characterisation

**Figure 1:**
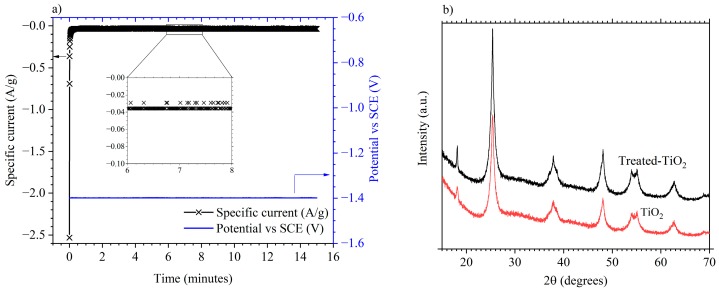
(**a**) Electrochemical reduction of TiO_2_ in 1 mol/dm^3^ KOH through a potentiostatic hold at −1.4 V vs. saturated calomel reference electrode (SCE); (**b**) XRD pattern from a TiO_2_ (bottom) and treated-TiO_2_ electrode (top).

The cathodic electrochemical treatment was carried out via a chronoamperometry performed on composite TiO_2_ electrodes. A potential of −1.40 V vs. SCE was applied to the electrode in 1 mol·dm^−3^ KOH in an attempt to partially reduce the TiO_2_ active material. The potential was stepped from 0.0 V vs. SCE to −1.40 V vs. SCE and held for 15 min. Figure 1a shows the resulting specific current vs. time profile. A cathodic current response of ca. 2.5 A/g decreases rapidly to ca. 0.10 A/g after 3 s and to less than 0.05 A/g after 7.8 s. Between minutes 6 and 8, the measured current is less than 0.04 A/g as shown by the inset of Figure 1a. The same electrochemical reduction protocol was also applied to electrodes without carbon black conductive additive. Figure 1b gives the XRD patterns from a TiO_2_ and treated-TiO_2_ electrode. Both show the characteristic anatase pattern with no difference between the two, suggesting a lack of bulk structural change to the TiO_2_ electrodes.

**Figure 2:**
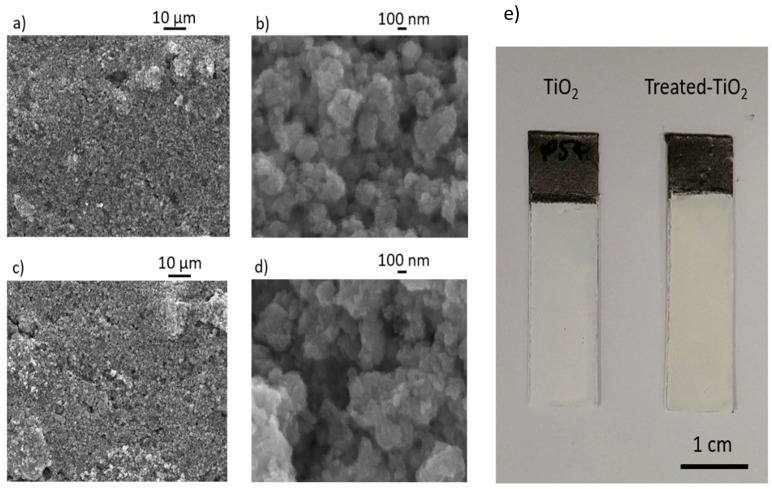
(**a**,**b**) present SEM images of a TiO_2_ electrode at 1000× and 4,0000× magnification; (**c**,**d**) show images from a treated-TiO_2_ electrode. (**e**) shows a photographic image of a TiO_2_ (left) and treated-TiO_2_ (right) electrode.

Figure 2a,b show SEM images of an as-manufactured TiO_2_ electrode, at 1000× and 40000× magnification, with Figure 2c–d giving images from treated-TiO_2_. At 1000× magnification, both electrodes show an even coating of active material, though larger agglomerations can also be seen. At 40000× magnification, individual particle agglomerations can be observed, though resolution of individual particles cannot be seen due to the small nominal TiO_2_ particle size of approximately 5 nm. No significant change can be observed in the structure of the electrodes, suggesting that any change in electrode performance was not due to particle flocculation or minor electrode cracking, which could have increased surface area or improved electrode wettability. Figure 2d shows photographic images of an as-manufactured (left) and electrochemically treated-TiO_2_ (right) electrode following the chronoamperometry shown in Figure 1a. Following treatment, the previously white TiO_2_ electrode can be seen to change colour to pale-yellow. This would be consistent with the introduction of Ti^3+^ and a change in the band gap of TiO_2_ [22,29]. Despite previous self-doped TiO_2_ nanotube arrays developing a blue colour [30], a yellow colour has also been shown to occur when TiO_2_ has undergone hydrogenation [31,32], hydrothermal treatment [33] or N_2_ doping [34].

**Figure 3:**
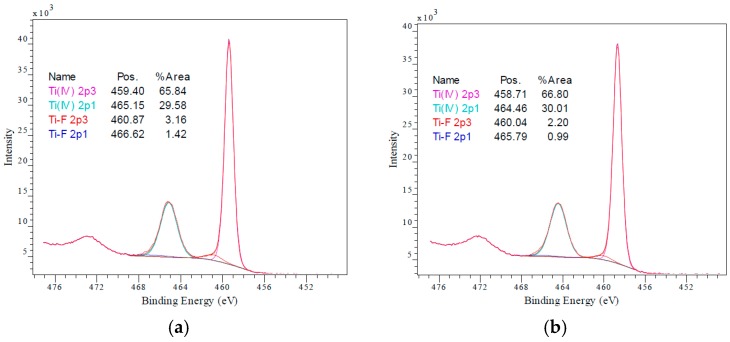
High resolution Ti 2p spectra of TiO_2_ (**a**) and treated-TiO_2_ (**b**) Original measurements given in red with brown denoting the sum of the fitted curves.

X-ray photoelectron spectroscopy was performed on the samples shown in Figure 2e to investigate the surface states of untreated (TiO_2_) and electrochemically treated TiO_2_ (treated-TiO_2_) electrodes. Figure 3 gives the Ti 2p spectra of TiO_2_ (a) and treated-TiO_2_ (b) electrodes. No detectable difference can be observed between the samples. This could indicate that Ti^3+^ states exist within the bulk rather than the surface. However, given that measurements were performed ex-situ, it is more likely that any Ti^3+^ formation, from the electrochemical reduction treatment, is followed by re-oxidation in air. Ti 2p_3/2_ peaks are located at 459.40 and 458.71 eV for TiO_2_ and treated-TiO_2_ respectively, while Ti 2p_1/2_ peaks are located at 465.15 and 464.46 eV. Unexpectedly, the presence of inorganic Ti-oxyfluoride < 1%, was detected in both samples, which may arise during ink preparation, but could alternatively arise from low level X-ray degradation of the binder within the ink.

**Figure 4:**
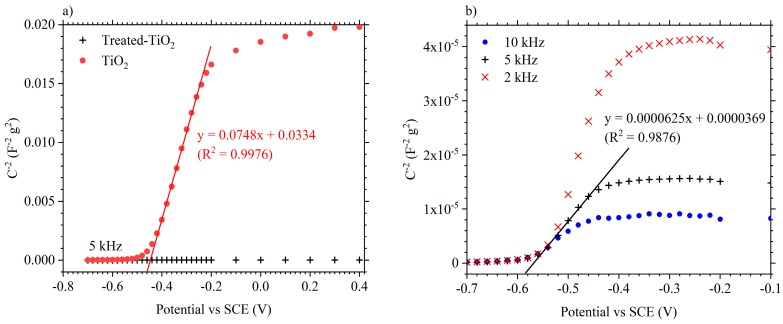
(**a**) Mott–Schottky plots of treated-TiO_2_ and TiO_2_ at 5 kHz in 1 mol·dm^−3^ AlCl_3_/1 mol·dm^−3^ KCl; (**b**) Mott–Schottky plots of the treated TiO_2_ electrode at 2, 5 and 10 kHz.

Figure 4 shows Mott–Schottky plots produced from EIS measurements performed at DC bias potentials between 0.4 V and −0.7 V vs. SCE, in 1 mol·dm^−3^ AlCl_3_/1 mol·dm^−3^ KCl. Figure 4a compares a TiO_2_ and treated-TiO_2_ electrode at a frequency of 5 kHz. Capacitance values were calculated via Equation (1), where *C* = capacitance, *f* = frequency and *Z*″ = imaginary impedance part. The positive and linear gradient produced by the as-manufactured TiO_2_ electrode, between approximately −0.45 V and −0.3 V vs. SCE, is indicative of an n-type semi-conductor and has an approximate gradient of 0.0748 [35,36]. The reduction in C^−2^ from the treated-TiO_2_ electrode suggests an increase in the number of donors (i.e., electrons) according to Equation 1, where *C* = capacitance, *A* = area, *N_d_* = number of donors, *e* = electronic charge, *V* = applied potential, *V_FB_* = flat band potential, *k* = Boltzmann’s constant and *T* = absolute temperature [35].

(1) $$C=-\frac{i}{2\pi fZ^{\prime \prime }}.$$

(2) $$\frac{1}{C^{2}}=\frac{2}{\varepsilon \varepsilon_{0}A^{2}eN_{d}}\left(V-V_{FB}-\frac{kT}{e}\right).$$

Figure 4b shows an expanded view of the response from treated-TiO_2_ and also shows the response at frequencies of 2 kHz and 10 kHz. The Mott–Schottky plot is again indicative of an n-type semi-conductor. The gradient of the linear portion of the plot, between −0.46 and −0.56 V vs. SCE, is approximately 6.25 × 10^−5^. This is 3 orders of magnitude lower than the approximate gradient found in the as-manufactured TiO_2_ electrode at the same frequency, providing clearer evidence that the electrochemical treatment resulted in an increase in the electron donor number. The number of donors, *N_d_*, was not estimated since the electrochemically active surface area was not known. Nevertheless, the cathodic electrochemical treatment resulted in an increase in the number of donors (electrons) and a shift of the *V_FB_* to a more negative potential. Both these criteria would be expected from an introduction of Ti^3+^ or oxygen vacancies into TiO_2_, as expected from the cathodic treatment at −1.4 V vs. SCE in 1 mol·dm^−3^ KOH.

### 3.2. Electrochemical Performance

**Figure 5:**
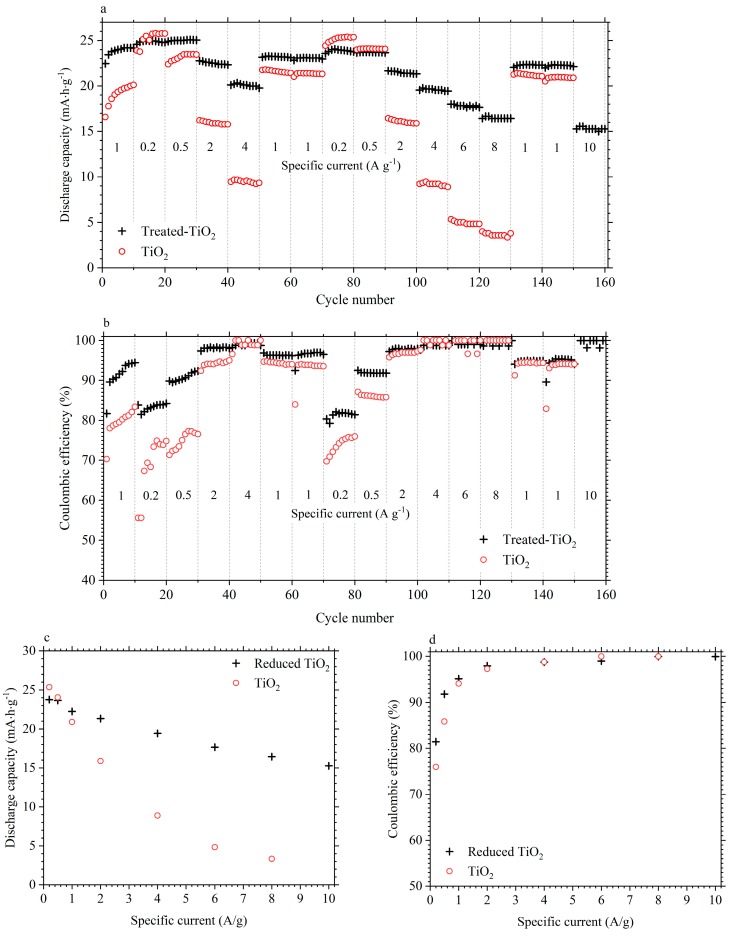
Discharge capacity (**a**) and coulombic efficiency (**b**) of a treated-TiO_2_ (black cross) and TiO_2_ electrode (red circle), at various specific currents, plotted against cycle number; (**c**) Compares the discharge capacity and (**d**) the coulombic efficiency at each specific current. Electrodes were cycled in 1 mol·dm^−3^ AlCl_3_/1 mol·dm^−3^ KCl between −1 V and +0.4 V vs. SCE.

To determine variations in electrode performance, 10 galvanostatic cycles were performed at each of the specific currents of 0.2, 0.5, 1.0, 2.0, 4.0, 6.0 and 8.0 A/g. Treated-TiO_2_ was then also cycled at 10.0 A/g. Figure 5a,b shows the discharge capacity and coulombic efficiency against the cycle number at the given specific currents. During the initial 10 cycles, the discharge capacity of both electrodes increases before stabilising during subsequent cycling, while it can be seen that the improved performance from treated-TiO_2_ becomes most obvious at higher specific currents. Figure 5b shows how the coulombic efficiency requires a greater number of cycles before stabilising. After 30 cycles, coulombic efficiency is relatively stable at a given specific current for both electrodes. To better show differences in performance, Figure 5c,d shows the discharge capacity and coulombic efficiency of the two electrodes as a function of specific current. The data points for Figure 5c,d were taken between cycle 60 and 160, shown in Figure 5a,b, using the 10th cycle from each specific current to ensure that a stable response had been reached. Figure 5c shows the discharge capacity of TiO_2_, at 0.2 A·g^−1^ to be 25.3 mA·h·g^−1^, which is actually marginally higher than the 23.8 mA·h·g^−1^ measured from the treated-TiO_2_. However, the improved performance of treated-TiO_2_ becomes obvious at higher specific currents. At 2 A/g, the discharge capacities of TiO_2_ and treated-TiO_2_ are 15.9 mA·h·g^−1^ and 21.3 mA·h·g^−1^ respectively. At 8 A/g, the TiO_2_ electrode can produce a capacity of only 3.2 mA·h·g^−1^ while a capacity of 15.3 mA·h·g^−1^ was measured from treated-TiO_2_ at the higher specific current of 10 A/g, clearly showing superior rate capability. The coulombic efficiency of treated-TiO_2_ was measured to be 81.4% and 91.8% at 0.2 A/g and 0.5 A/g, respectively. This is higher than a coulombic efficiency of 75.9% and 85.0% for the TiO_2_ electrode. Above specific currents of 1.0 A/g, the coulombic efficiency of both electrodes are roughly similar, increasing from approximately 98% to > 99.95% at 8.0 A/g. 

**Figure 6:**
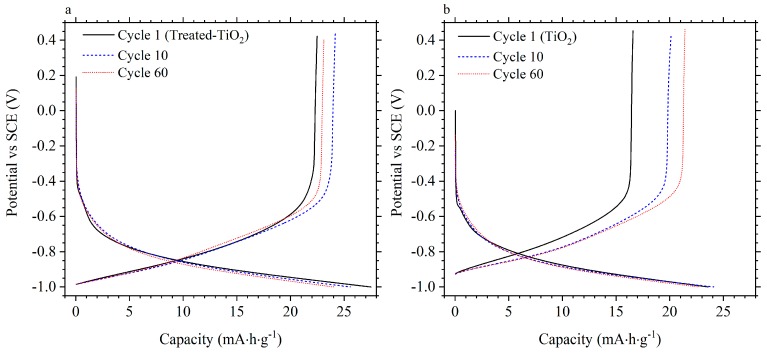
Voltage profile of the treated-TiO_2_ (**a**) and TiO_2_ (**b**) electrode during cycling at 1 A/g in 1 mol·dm^−3^ AlCl_3_/1 mol·dm^−3^ KCl at cycles 1, 10 and 60.

Figure 6 gives the voltage profiles, at 1 A/g, of the same treated−TiO_2_ (a) and TiO_2_ (b) electrodes during the 1st, 10th and 60th cycle. For treated-TiO_2_, a 1st cycle discharge capacity of 22.5 mA·h·g^−1^ was measured, corresponding to a coulombic efficiency of 81.7%. This increased to 24.2 mA·h·g^−1^ at a coulombic efficiency of 94.44% by the 10th cycle with a discharge capacity and coulombic efficiency of 23.1 mA·h·g^−1^ and 96.2% measured during the 60th cycle. For the untreated TiO_2_ electrode, an initial discharge capacity of 16.6 mA·h·g^−1^ was measured with a coulombic efficiency of 70.30%. Discharge capacity and coulombic efficiency were measured as 20.1 mA·h·g^−1^ and 83.4% during the 10th cycle and 21.4 mA·h·g^−1^ and 94.0% by the 60th cycle. The increased discharge capacity and coulombic efficiency from the treated-TiO_2_ electrode during initial cycling would be a particularly important consideration for full cell construction.

**Figure 7:**
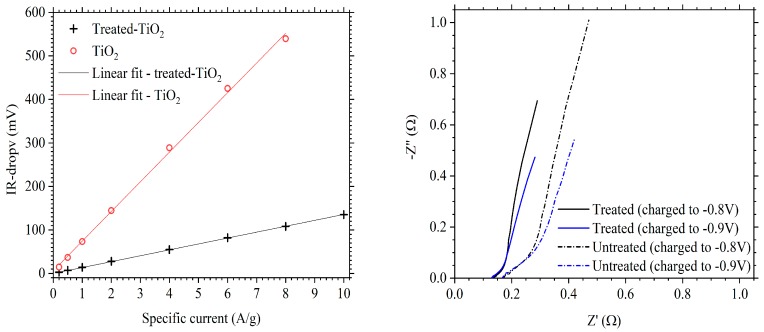
(**a**) Comparison of IR-drop, between charge and discharge, for treated-TiO_2_ (black cross) and TiO_2_ (red circle) as a function of specific current; (**b**) Nyquist plot of a TiO_2_ (dash-dot line) and treated-TiO_2_ (solid line) electrode. Measurements were taken between 0.1 Hz–10 kHz, at open circuit potential (OCP), after charging to −0.8 V (black) and −0.9 V (blue) vs. SCE.

The IR-drop, between charge and discharge, was also observed to be lower for the treated-TiO_2_ electrode throughout the cycling regime, resulting in a lower equivalent series resistance (ESR) for the electrochemically treated electrode. This can be seen in Figure 7a. Both electrodes follow a linear increase in IR-drop with increasing specific current as represented by the linear fits with x and y intercepts of 0. The difference between the treated-TiO_2_ and TiO_2_ electrodes is 11.7 mV at 0.2 A/g, increasing linearly to 116.5 mV at 2 A/g. At 10.0 A/g, the IR-drop from treated-TiO_2_ is 135.2 mV, while the IR-drop from TiO_2_ at 8.0 A/g is 539.3 mV. The gradient of the linear fits were used to determine an ESR of 0.562 Ω for treated-TiO_2_ and 2.843 Ω for untreated TiO_2_. This suggests a significant improvement in the conductivity of TiO_2_, a reasonable conclusion from the introduction of Ti^3+^ expected from the electrochemical reduction process, which would decrease the band gap and thus improve conductivity. Electrodes were 23 and 24 mg for the treated and pristine electrodes respectively, both covering an area of 7.0 cm^−2^ such that differences in electrode impedance arising from differences in mass loading or electrode thickness can be reasonably neglected.

The effect of the treatment was further characterised with the use of electrochemical impedance spectroscopy (EIS). TiO_2_ and treated-TiO_2_ electrodes were left at open circuit potential (OCP) for 1 h before being subject to EIS protocols. Measurements were performed between 0.1 Hz–10 kHz with a 20 mV perturbation. Electrodes were analysed in partially charged states by applying a 100 mA/g charge current to −0.8 V and −0.9 V, holding the electrodes at these potentials for 120 s then performing measurements at OCP. Potentials of 0.8 V and 0.9 V vs. SCE correspond to states-of-charge of approximately 20% and 50%, respectively. Figure 7b gives the Nyquist plot of a pristine and electrochemically treated TiO_2_ electrode at OCP after charging to −0.8 V or −0.9 V For both electrodes, the Nyquist plots follow a ca. 45° impedance, starting at 0.13 Ω on the real x-axis for treated-TiO_2_ and 0.17 Ω for TiO_2_ due to the series resistance arising from the electrolyte and electrode. This 45° line curves up to > 45° at a frequency of approximately 2 Hz for the treated electrode and at approximately 1 Hz for the as-manufactured electrode. The characteristic semi-circle present for many insertion battery electrodes is not immediately obvious, suggesting either a rapid charge-transfer or a very small charge-transfer resistance; this suggests that charge storage is diffusion limited. The > 45° slope present at mid to low frequencies further suggest the possibility of capacitive behaviour from the electrodes [28], though the plateau observed during galvanostatic cycling indicates a faradaic mechanism being responsible for charge capacity. The lower length of the segment in the high to mid frequency region, of treated-TiO_2_ compared to untreated TiO_2_, suggests a lower impedance. This could be assigned to a lower charge transfer resistance if the high frequency region is represented by a depressed semi-circle with a smaller radius. Alternatively, the ca. 45° line may represent a Warburg impedance, which can describe the transport of ions through electrode pores [37].

**Figure 8:**
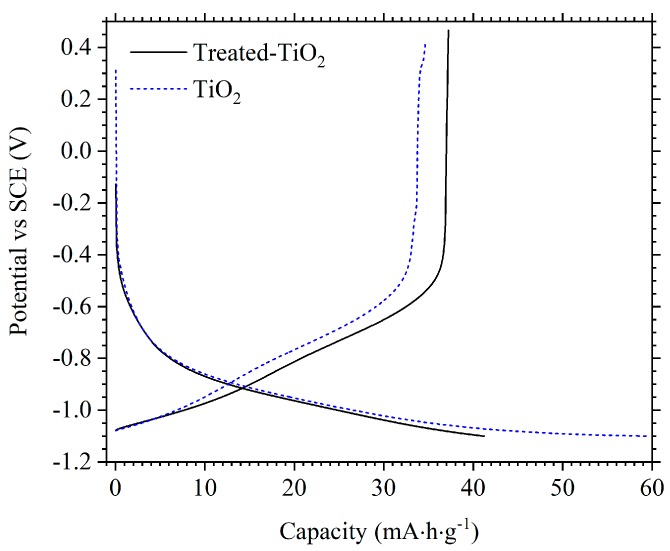
Voltage profiles of TiO_2_ (blue-dash) and treated-TiO_2_ (black) cycled to −1.1 V vs. SCE at 1.0 A/g.

Furthermore, TiO_2_ and treated-TiO_2_ were cycled to −1.1 V vs SCE. Figure 8 shows the voltage profile of TiO_2_ and treated-TiO_2_ cycled at 1 A/g to −1.1 V. An increase in discharge capacity is measured from both electrodes: 34.6 mA·h·g^−1^ and 37.2 mA·h·g^−1^ for TiO_2_ and treated-TiO_2_ respectively. However, there is a considerable decrease in coulombic efficiency from TiO_2_, measured at only 58.0% compared to 85.0% when cycled to −1.0 V at 1 A/g. In contrast, coulombic efficiency is only marginally lower for treated-TiO_2_ at 90.3% compared to 90.9% when cycled to −1.0 V. This further demonstrates the drastic improvement provided by the electrochemical reduction treatment.

**Figure 9:**
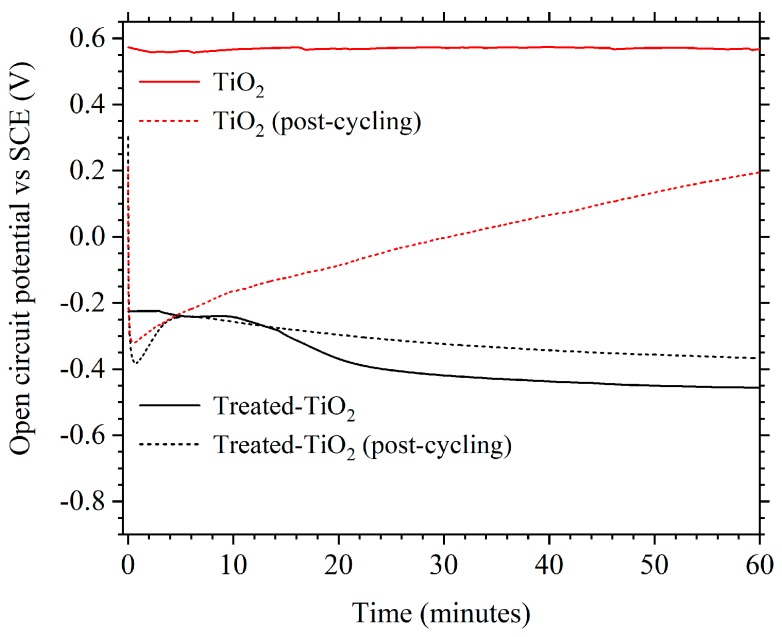
The 1 h OCP measurements of TiO_2_ (red) and treated-TiO_2_ (black) taken before cycling (dashed-line) and after the cycling regime presented in Figure 5 and Figure 6 (solid-line).

It was also observed that the OCP of treated-TiO_2_ was altered compared to TiO_2_, both before and after cycling. Figure 9 shows that before cycling, treated-TiO_2_ has an OCP of approximately −0.425 V vs. SCE with the OCP of TiO_2_ being approximately 0.58 V. Post-cycling, the OCP of treated-TiO_2_ after 1 h is relatively stable at approximately −0.38 V, with TiO_2_ being at 0.2 V and still increasing after 1 h at OCP. This implies a change in the equilibrium potential and therefore a change in the concentration of some species within the electrode, which could be the incorporation of Ti^3+^ due to the electrochemical treatment. It cannot be ruled out that the electrochemical reduction process improves performance through factors such as improved electrode wetting or enhanced surface area, though the lack of structural changes observed via SEM imaging and XRD suggest otherwise. However, while XPS showed that there was no measurable change in oxidation state, the observed colour change, different OCP values and Mott-Schottky plots suggest a change in the band gap and charge carrier density of TiO_2_, which would be consistent with the introduction of Ti^3+^. This change in OCP also remains stable in the electrolyte and during cycling. 

## 4. Conclusions

Electrochemically treated TiO_2_ electrodes were prepared through a potentiostatic hold at −1.4 V vs. SCE, in 1 mol·dm^−3^ KOH. The colour change of a TiO_2_ electrode from white to pale yellow suggests the introduction of Ti^3+^, though this could not be confirmed with XPS measurements. However, a Mott–Schottky plot showed treated-TiO_2_ to have a greater electron donor number, which would be the expected result from the introduction of Ti^3+^ or oxygen vacancies. The electrochemical reduction protocol was shown to improve capacity, coulombic efficiency and stability during the first 60 galvanostatic cycles. Conductivity also drastically improved, observed via EIS and analysis of the IR-drop between charge and discharge cycles. It is demonstrated that the electrochemical treatment described can improve the performance of composite TiO_2_ electrodes for use in aqueous Al-ion batteries and could also be applied to titanium oxide electrodes in other metal-ion batteries. A discharge capacity of 15.3 mA·h·g^−1^ was possible from treated-TiO_2_ at 10 A/g—the highest specific current recorded from TiO_2_ in aqueous Al^3+^ electrolyte and a higher specific current than recorded from NaTi_2_(PO_4_)_3_ in aqueous Na-ion cells. The observed improvements to coulombic efficiency and stability during the first 10 cycles are particularly important for use in full cells, but still requires further improvements and understanding. Cycling a separate treated-TiO_2_ electrode at 1 A/g allowed for a capacity of 37.2 mA·h·g^−1^ at 90.3% coulombic efficiency, where an as-manufactured TiO_2_ electrode produced 34.6 mA·h·g^−1^ at a much lower coulombic efficiency of 58.0%, further demonstrating the improved stability from the electrochemically treated TiO_2_ electrodes. This work also highlights the importance of TiO_2_ doping for improved electrode performance in high-rate, aqueous electrolyte batteries.

## References

[1] Wang Y., Feng Z., Laul D., Zhu W., Provencher M., Trudeau M.L., Guerfi A., Zaghib K. (2018). Ultra-low cost and highly stable hydrated FePO4 anodes for aqueous sodium-ion battery. J. Power Sources.

[2] Li W., Dahn J.R., Wainwright D.S. (1994). Rechargeable Lithium Batteries with Aqueous Electrolytes. Science.

[3] Kim H., Hong J., Park K.-Y., Kim H., Kim S.-W., Kang K. (2014). Aqueous Rechargeable Li and Na Ion Batteries. Chem. Rev..

[4] Wang L.-P., Wang P.-F., Wang T.-S., Yin Y.-X., Guo Y.-G., Wang C.-R. (2017). Prussian blue nanocubes as cathode materials for aqueous Na-Zn hybrid batteries. J. Power Sources.

[5] Holland A., Mckerracher R.D., Cruden A., Wills R.G.A. (2018). An aluminium battery operating with an aqueous electrolyte. J. Appl. Electrochem..

[6] Kazazi M., Abdollahi P., Mirzaei-Moghadam M. (2017). High surface area TiO_2_ nanospheres as a high-rate anode material for aqueous aluminium-ion batteries. Solid State Ion..

[7] He Y.J., Peng J.F., Chu W., Li Y.Z., Tong D.G. (2014). Black mesoporous anatase TiO_2_ nanoleaves: A high capacity and high rate anode for aqueous Al-ion batteries. J. Mater. Chem. A.

[8] Lahan H., Boruah R., Hazarika A., Das S.K. (2017). Anatase TiO_2_ as an Anode Material for Rechargeable Aqueous Aluminum-Ion Batteries: Remarkable Graphene Induced Aluminum Ion Storage Phenomenon. J. Phys. Chem. C.

[9] Liu Y., Sang S., Wu Q., Lu Z., Liu K., Liu H. (2014). The electrochemical behavior of Cl^−^ assisted Al^3+^ insertion into titanium dioxide nanotube arrays in aqueous solution for aluminum ion batteries. Electrochim. Acta.

[10] Liu S., Hu J.J., Yan N.F., Pan G.L., Li G.R., Gao X.P. (2012). Aluminum storage behavior of anatase TiO_2_ nanotube arrays in aqueous solution for aluminum ion batteries. Energy Environ. Sci..

[11] Holland A.W., McKerracher R., Cruden A., Wills R.G.A. (2018). TiO_2_ nanopowder as a high rate, long cycle life electrode in aqueous aluminium electrolyte. Mater. Today Energy.

[12] Wang H., Zhang T., Chen C., Ling M., Lin Z., Zhang S., Pan F., Liang C. (2018). High-performance aqueous symmetric sodium-ion battery using NASICON-structured Na_2_VTi(PO_4_)_3_. Nano Res..

[13] Deng C., Zhang S., Dong Z., Shang Y. (2014). 1D nanostructured sodium vanadium oxide as a novel anode material for aqueous sodium ion batteries. Nano Energy.

[14] Whitacre J.F., Wiley T., Shanbhag S., Wenzhuo Y., Mohamed A., Chun S.E., Weber E., Blackwood D., Lynch-Bell E., Gulakowski J. (2012). An aqueous electrolyte, sodium ion functional, large format energy storage device for stationary applications. J. Power Sources.

[15] Zhang B.H., Liu Y., Chang Z., Yang Y.Q., Wen Z.B., Wu Y.P., Holze R. (2014). Nanowire Na_0.35_MnO_2_ from a hydrothermal method as a cathode material for aqueous asymmetric supercapacitors. J. Power Sources.

[16] Gang P., Ping N., Changzhou Y., Laifa S., Xiaogang Z., Jiajia Z., Bing D. (2014). Enhanced Performance of Aqueous Sodium-Ion Batteries Using Electrodes Based on the NaTi_2_(PO_4_)_3_/MWNTs–Na_0.44_MnO_2_ System. Energy Technol..

[17] Liang C., Leyuan Z., Xufeng Z., Zhaoping L. (2014). Aqueous Batteries Based on Mixed Monovalence Metal Ions: A New Battery Family. ChemSusChem.

[18] Yao H.-R., You Y., Yin Y.-X., Wan L.-J., Guo Y.-G. (2016). Rechargeable dual-metal-ion batteries for advanced energy storage. Phys. Chem. Chem. Phys..

[19] Lu K., Song B., Zhang J., Ma H. (2016). A rechargeable Na-Zn hybrid aqueous battery fabricated with nickel hexacyanoferrate and nanostructured zinc. J. Power Sources.

[20] Takai O. (2008). Solution plasma processing (SPP). Pure Appl. Chem..

[21] Pitchaimuthu S., Honda K., Suzuki S., Naito A., Suzuki N., Katsumata K.-I., Nakata K., Ishida N., Kitamura N., Idemoto Y. (2018). Solution Plasma Process-Derived Defect-Induced Heterophase Anatase/Brookite TiO_2_ Nanocrystals for Enhanced Gaseous Photocatalytic Performance. ACS Omega.

[22] Chen X., Liu L., Huang F. (2015). Black titanium dioxide (TiO_2_) nanomaterials. Chem. Soc. Rev..

[23] Lu X., Wang G., Zhai T., Yu M., Gan J., Tong Y., Li Y. (2012). Hydrogenated TiO_2_ Nanotube Arrays for Supercapacitors. Nano Lett..

[24] Qiu J., Li S., Gray E., Liu H., Gu Q.-F., Sun C., Lai C., Zhao H., Zhang S. (2014). Hydrogenation Synthesis of Blue TiO_2_ for High-Performance Lithium-Ion Batteries. J. Phys. Chem. C.

[25] Shin J.-Y., Joo J.H., Samuelis D., Maier J. (2012). Oxygen-Deficient TiO_2−δ_ Nanoparticles via Hydrogen Reduction for High Rate Capability Lithium Batteries. Chem. Mater..

[26] He H., Yang K., Wang N., Luo F., Chen H. (2013). Hydrogenated TiO_2_ film for enhancing photovoltaic properties of solar cells and self-sensitized effect. J. Appl. Phys..

[27] Song J., Zheng M., Yuan X., Li Q., Wang F., Ma L., You Y., Liu S., Liu P., Jiang D. (2017). Electrochemically induced Ti^3+^ self-doping of TiO_2_ nanotube arrays for improved photoelectrochemical water splitting. J. Mater. Sci..

[28] Zhou H., Zhang Y. (2014). Electrochemically Self-Doped TiO_2_ Nanotube Arrays for Supercapacitors. J. Phys. Chem. C.

[29] Nah Y.-C., Paramasivam I., Schmuki P. (2010). Doped TiO_2_ and TiO_2_ Nanotubes: Synthesis and Applications. ChemPhysChem.

[30] Zhou Y., Chen C., Wang N., Li Y., Ding H. (2016). Stable Ti^3+^ Self-Doped Anatase-Rutile Mixed TiO_2_ with Enhanced Visible Light Utilization and Durability. J. Phys. Chem. C.

[31] Lu H., Zhao B., Pan R., Yao J., Qiu J., Luo L., Liu Y. (2014). Safe and facile hydrogenation of commercial Degussa P25 at room temperature with enhanced photocatalytic activity. RSC Adv..

[32] Leshuk T., Parviz R., Everett P., Krishnakumar H., Varin R.A., Gu F. (2013). Photocatalytic Activity of Hydrogenated TiO_2_. ACS Appl. Mater. Interfaces.

[33] Ren R., Wen Z., Cui S., Hou Y., Guo X., Chen J. (2015). Controllable Synthesis and Tunable Photocatalytic Properties of Ti^3+^-doped TiO_2_. Sci. Rep..

[34] Wang W., Ni Y., Lu C., Xu Z. (2014). Hydrogenation temperature related inner structures and visible-light-driven photocatalysis of N–F co-doped TiO_2_ nanosheets. Appl. Surf. Sci..

[35] Gelderman K., Lee L., Donne S.W. (2007). Flat-Band Potential of a Semiconductor: Using the Mott–Schottky Equation. J. Chem. Educ..

[36] Sellers M.C.K., Seebauer E.G. (2011). Measurement method for carrier concentration in TiO_2_ via the Mott–Schottky approach. Thin Solid Films.

[37] Yadav P., Pandey K., Bhatt P., Tripathi B., Pandey M.K., Kumar M. (2016). Probing the electrochemical properties of TiO_2_/graphene composite by cyclic voltammetry and impedance spectroscopy. Mater. Sci. Eng. B.

